# A Comparative Study for the Evaluation of Two Doses of Ellagic Acid on Hepatic Drug Metabolizing and Antioxidant Enzymes in the Rat

**DOI:** 10.1155/2013/358945

**Published:** 2013-07-18

**Authors:** Gurbet Celik, Aslı Semiz, Serdar Karakurt, Sevki Arslan, Orhan Adali, Alaattin Sen

**Affiliations:** ^1^Department of Biology, Pamukkale University, Kinikli Campus, 20070 Denizli, Turkey; ^2^Department of Biochemistry, Institute of Natural and Applied Science, Middle East Technical University, 06800 Ankara, Turkey; ^3^Faculty of Art & Sciences, Biology Department, Pamukkale University, Kinikli, 20070 Denizli, Turkey

## Abstract

The present study was designed to evaluate different doses of ellagic acid (EA) *in vivo* in rats for its potential to modulate hepatic phases I, II, and antioxidant enzymes. EA (10 or 30 mg/kg/day, intragastrically) was administered for 14 consecutive days, and activity, protein, and mRNA levels were determined. Although the cytochrome P450 (CYP) 2B and CYP2E enzyme activities were decreased significantly, the activities of all other enzymes were unchanged with the 10 mg/kg/day EA. In addition, western-blot and qRT-PCR results clearly corroborated the above enzyme expressions. On the other hand, while the NAD(P)H:quinone oxidoreductase 1 (NQO1), catalase (CAT), glutathione peroxidase (GPX), and glutathione S-transferase (GST) activities were increased significantly, CYP1A, 2B, 2C, 2E, and 19 enzyme activities were reduced significantly with 30 mg/kg/day EA. In addition, CYP2B, 2C6, 2E1, and 19 protein and mRNA levels were substantially decreased by the 30 mg/kg/day dose of EA, but the CYP1A protein, and mRNA levels were not changed. CYP3A enzyme activity, protein and mRNA levels were not altered by neither 10 nor 30 mg/kg/day ellagic acid. These results indicate that EA exerts a dose-dependent impact on the metabolism of chemical carcinogens and drugs by affecting the enzymes involved in xenobiotics activation/detoxification and antioxidant pathways.

## 1. Introduction 

Ellagic acid (EA) is one of the important components of fruits and vegetables [1] and has been shown to possess numerous anticarcinogenic and antimutagenic properties towards various carcinogens [1–7]. Several mechanisms have been proposed to explain the broad antimutagenic and anticarcinogenic effects of EA [8–13]. One of the mechanisms proposed involves inhibition of cytochrome P450 (CYP450) enzymes [8]. CYP450 enzymes are widely known for their role in the metabolism of drugs and other foreign compounds. Thus, modulation of this enzyme system can influence the metabolism of xenobiotics, producing effects of pharmacological and toxicological importance. A number of naturally occurring flavonoids have been shown to modulate the CYP450 system by the activation or inhibition of these enzymes [14]. Multiple P450 isozymes show different substrate specificities and affinities toward both endogenous and exogenous compounds. Among these P450s, CYP1A, 2B, 2C, 2E, and 3A subfamilies have received a great deal of attention in recent years because of their ability to metabolize various pharmaceutical and carcinogenic agents [15–17]. It is well established that CYP1A and CYP2E enzymes are mainly involved in carcinogen metabolism while CYP3A, CYP2B, and CYP2C enzymes are mainly responsible for drug metabolism.

Aromatase represents a crucial enzyme of estrogen biosynthesis, and increased expression of aromatase has been observed in breast cancer tissue [18]. Flavonoids and isoflavones are structurally similar to the endogenous estradiol and possess both estrogenic and antiestrogenic activities [19]. Previous studies suggest that ellagic acid has potential for the prevention of estrogen-responsive breast cancers [20, 21].

Another possible mechanism for the broad chemoprotective and antioxidant effects of EA might involve the induction of phase II and antioxidant enzymes. GSTs have a considerably important role in the detoxification of carcinogens [22]. NQO1 prevents quinine redox cycling and lowers levels of electrophilic quinines [23]. Hence, the inductions of GST and NQO1 by flavonoids are possibly associated with cancer chemopreventive effects. The present study is, to our knowledge, the first study analyzing the effect of EA on NQO1 activity, mRNA, and protein levels in rat.

Although there are individual studies examining the effect of EA on several enzymes, no study has evaluated the alteration of so many enzymes simultaneously with different dose of EA. Therefore, the present study was undertaken to observe the overall simultaneous changes following *in vivo* treatment with different dose of EA by investigating changes in the activity, mRNA, and protein levels of specific rat hepatic P450s, phase II, and antioxidant enzymes at once.

## 2. Material and Methods

### 2.1. Chemicals

The following chemicals were purchased from Sigma-Aldrich Chemical Company (St Louis, Missouri, USA): ellagic acid, acrylamide, aniline, anti-rabbit IgG-HRP conjugate, bovine serum albumin (BSA), Folin phenol reagent, glycerol, glycine, HEPES, *β*-NADPH, phenol, caffeine, TRIS, PMSF, potassium dihydrogen phosphate, dipotassium hydrogen phosphate, sodium dodecyl sulfate (SDS), and sodium potassium tartrate. Anti-rat CYP1A1, CYP2B, CYP2C6, CYP2E1, CYP3A1, and CYP19 antibodies were from Abcam (Abcam PLC, Cambridge, UK). All other chemicals and solvents were obtained from commercial sources at the highest grade of purity available.

### 2.2. Animals and Treatment

Healthy male Wistar rats, about 12 weeks old and weighing 200–250 g, were obtained from the University Animal House. They were housed in small cages at an ambient temperature of 22 ± 1°C, on a 12 h light/dark cycle, and a standard pellet diet and distilled water were available without restriction. All experimental procedures with the animals were performed under appropriate regimes with veterinary services within the licensed projects (PAUHADEK-2009-007).

After being acclimatized for 1 week, the rats were randomized and divided into three groups. Ellagic acid was given intragastrically to two experimental groups (15 rats per group) in a dose of 10 mg/kg/day (T10) and 30 mg/kg/day (T30) dissolved in DMSO, respectively. Control (15 rats) rats only received a vehicle (DMSO). The doses used were chosen to be similar to the doses used in previous studies in the literature [24–26]. The animals were treated for 14 consecutive days. At the end of the experimental period and following 16 h of fasting, blood was collected by heart puncture and the rats were killed; the livers were removed, rinsed with cold physiological saline, and stored at −80°C until analyzed.

### 2.3. Preparations of S1.5, Cytosolic, and Microsomal Fraction

Tissues were homogenized in a 4 part homogenization solution (1.15% KCl containing 3 mM EDTA, 0.5 mM APMSF, 0.3 mM *ε*-aminocaproic acid, 0.15 mM butylated hydroxytoluene, and 0.025% Triton X-100) using a tissue homogenizer with a Teflon pestle at 4°C. Subcellular fractions (S1.5, cytosolic, microsomal) of rat tissues were prepared by standard differential centrifugation with calcium aggregation as described by [27]. The amount of protein in individual fractions was measured using BCA [28] with BSA as the standard.

### 2.4. Enzyme Assays

Serum aspartate aminotransferase (AST) and alanine aminotransferase (ALT) activities were determined with an autoanalyzer using audit diagnostics AST and ALT. The microsomal cytochrome P450-dependent aniline 4-hydroxylase (A4H) activities of rat microsomes were determined by measuring the quantity of p-aminophenol formed, as described by [29]. Aminopyrene N-demethylase (APND), erythromycin N-demethylase (ERND) and caffeine N-demethylase (C3ND), n-nitrosodimethylamine N-demethylase (NDMA), ethylmorphine N-demethylase (EmND), and benzphetamine N-demethylase (BPND) activities were determined by measuring the quantity of formaldehyde formed, according to the method of [30] and modified by [31]. Ethoxyresorufin O-deethylase (EROD), and methoxyresorufin *O*-demethylase (MROD), benzyloxyresorufin *O*-demethylase (BROD), pentoxyresorufin *O*-demethylase (PROD) activities were assayed as described by [15]. Dibenzylfluorescein-O-debenzylase (DBFOD) activities were assayed as described by [32]. Rat liver NQO1 enzyme activity was determined according to the method of [33]. Glutathione *S*-transferase (GST) activities were assayed as described by [34]. Catalase (CAT) and glutathione peroxidase (GPx) activities were determined by [35, 36], respectively.

### 2.5. Gel Electrophoresis and Western Blotting

SDS-PAGE and western blotting were performed as described previously [15]. Briefly, 120 *μ*g protein samples were separated on 8.5% polyacrylamide gels using the discontinuous buffer system of [37]. Proteins were transferred to a nitrocellulose membrane by the iBlot dry blotting system (20 V, 12 min), using iBlot gel transfer stacks. Following transfer, the membranes were blocked using 5% nonfat dry milk in TBST (20 mM Tris-HCl, pH 7.4, 400 mM NaCl, and 0.1% (v/v) Tween20) for 60 min, and incubated with mouse polyclonal anti-rat CYP1A1, CYP2B, CYP2C6, CYP2E1, CYP3A1, or CYP19 antibodies (diluted 1 : 1000 in blocking solution) for 120 min at room temperature. The membranes were then washed with TBST (3 × 5 min), incubated with the secondary antibody (HRP-conjugated anti-rabbit IgG at a 1 : 5000 or 1 : 10000 dilution) for 60 min and again washed with TBST (3 × 5 min). Proteins were detected using SuperSignal West Pico Chemoluminescent Substrate (Pierce, Rockford, IL, USA), and bands were visualized and recorded using GelQuant Image Analysis Software in a DNR LightBIS Pro Image Analysis System (DNR Bio-Imaging Systems Ltd., Jerusalem, Israel). Protein bands were quantified using Scion Image Version Beta 4.0.2 software.

### 2.6. RNA Isolation and qRT-PCR of CYP mRNAs

Total RNA was extracted from 100 mg rat livers using TRIzol reagent. Extracted RNA was quantified spectrophotometrically at 260/280 nm, and the integrity was checked using 1% agarose gel. For cDNA synthesis, 2.5 *μ*g of RNA was incubated at 70°C for 10 minutes with 0.5 *μ*g of oligo (dT). After 5 min on ice, 50 U Moloney murine leukemia virus reverses transcriptase, 1 mM dNTPs and 5X reaction buffer were added to the previous mixture and incubated at 42°C for 60 min. The reaction was stopped by heating the mixture to 70°C for 10 min, and the cDNA was stored at −80°C for further use.

qRT-PCR assay was performed by using gene specific primers. The oligosequences used as forward and reverse primers for rat CYP450 isozymes were based on those reported in [38, 39]. Glyceraldehyde-3-phosphate dehydrogenase (GAPDH) and *β*-actin were used as a housekeeping gene. The PCR amplification was done using Power SYBR Green PCR master mix (Roche Applied Science, Basel, Switzerland) and 500 nmol/L of forward and reverse primers for each gene, for which the final primer concentration was 125 nmol/L each. Quantitative PCR was done using a Light Cycler 1.5 Instrument (Roche Applied Science, Basel, Switzerland). The PCR conditions were as follows: DNA polymerase activation at 95°C for 15 minutes, followed by 45 cycles at 95°C for 10 seconds, 54–57°C annealing (depending on the gene) for 5 seconds, and 72°C for 30 seconds. All gene analyses were done at least three times. 

### 2.7. Statistical Analysis

Statistical analyses were performed using the Minitab 13 statistical software package (Minitab, Inc., State College, PA, USA). All results were expressed as means including their Standard Error of Means (SEMs). Comparison between groups was performed using Student's *t*-test, and *P* < 0.05 was selected as the level required for statistical significance. Statistical comparisons between three groups were assessed by one-way analysis of variance (ANOVA). When *F* ratios were significant (*P* < 0.05), one-way ANOVA was followed by Tukey's Post hoc test for comparisons of multiple group means.

## 3. Results

Control and treated rats showed no significant differences in food consumption or body weight, (data not shown) after intragastric delivery of the EA to the animals at two different doses (T10 and T30).

As shown in Table 1, blood serum AST and ALT activities were not changed when compared to the control rats. As presented in Table 2, hepatic 1A1-associated EROD and 1A2-associated MROD or C3ND activities were decreased 11% and 13% or 40% in T30 treated rats, respectively, when compared to controls. 

The effect of the EA on CYP2B-associated BPND, EmND, BROD, and PROD activities is presented in Table 2. EA treatment at T10 or T30 for 14 consecutive days caused a statistically significant (*P* < 0.05) 13%, 10%, 15%, and 10% or 55%, 38%, 23%, and 30% decrease in these activities in the liver, respectively. 

CYP2E-associated A4H and NDMA activities in control and EA-treated rats are given in Table 2. As can be seen, A4H and NDMA activities were reduced 58% and 56% with the T30 treatment. Similarly, EA treatment at T10 reduced 28% and 26% with respect to the control.

The effect of the EA on CYP2C6-associated APND, CYP3A-associated ERND, and CYP19-associated DBFOD activity is presented in Table 2. EA treatment at T30 caused a statistically significant 55%, 28%, and 24% decrease in CYP2C6, CYP3A, and CYP19 activities in the liver, respectively. 

GSTs activities in control and EA-treated rats are given in Table 2. GST activities towards three different substrates, namely, CDNB, DCNB, and EA were increased 49%, 42%, and 33% (*P* < 0.05) in the T30 treated group. Furthermore, treatment of rats with T30 dose caused 194%, 64%, and 114% increases in NQO1, CAT, and GPX activities, respectively, when compared to the control values (Table 2).

**Figure 1:**
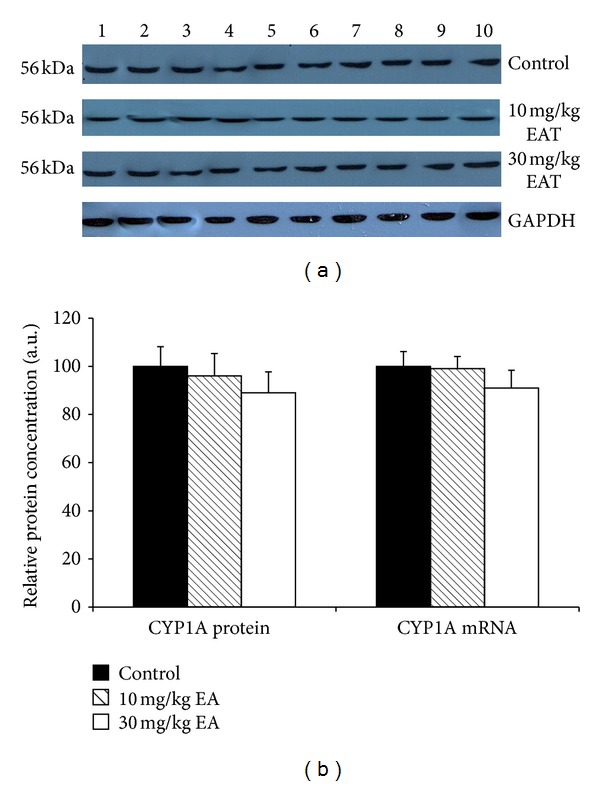
The expression levels of CYP1A protein and mRNA in control rats and rats treated with EA. Treatments were carried out as described in Section 2. Lanes 1–10, rat liver microsome sample. Wells have equal amount of protein. (a) Representative immunoblot analysis of liver microsomal CYP1A proteins in sample and experimental groups, using rabbit anti-rat CYP1A IgG for 1 h at room temperature. Proteins were detected using chemoluminescent substrate for 3 minutes, and bands were visualized and recorded using a DNR LightBIS Pro Image Analysis System. (b) Comparison of CYP1A protein and mRNA levels among experimental groups. The bar graphs represent the mean intensity of the bands obtained from western blot and/or qRT-PCR results. Results are presented as the mean from three independent experiments and expressed as relative mean ± standard deviation.

**Figure 2:**
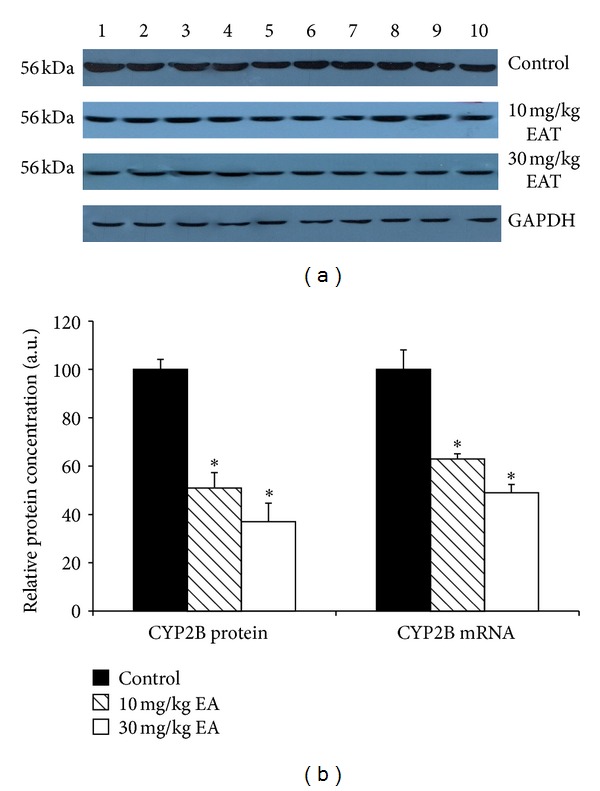
The expression levels of CYP2B protein and mRNA in control rats and rats treated with EA. Treatments were carried out as described in Section 2. Lanes 1–10, rat liver microsome sample. Wells have equal amount of protein. (a) Representative immunoblot analysis of liver microsomal CYP2B proteins in sample and experimental groups, using rabbit anti-rat CYP2B IgG for 1 h at room temperature. Proteins were detected using chemoluminescent substrate for 3 minutes, and bands were visualized and recorded using a DNR LightBIS Pro Image Analysis System. (b) Comparison of CYP2B protein and mRNA levels among experimental groups. The bar graphs represent the mean intensity of the bands obtained from western blot and/or qRT-PCR results. Results are presented as the mean from three independent experiments and expressed as relative mean ± standard deviation. *P < 0.05, compared with the control group.

**Figure 3:**
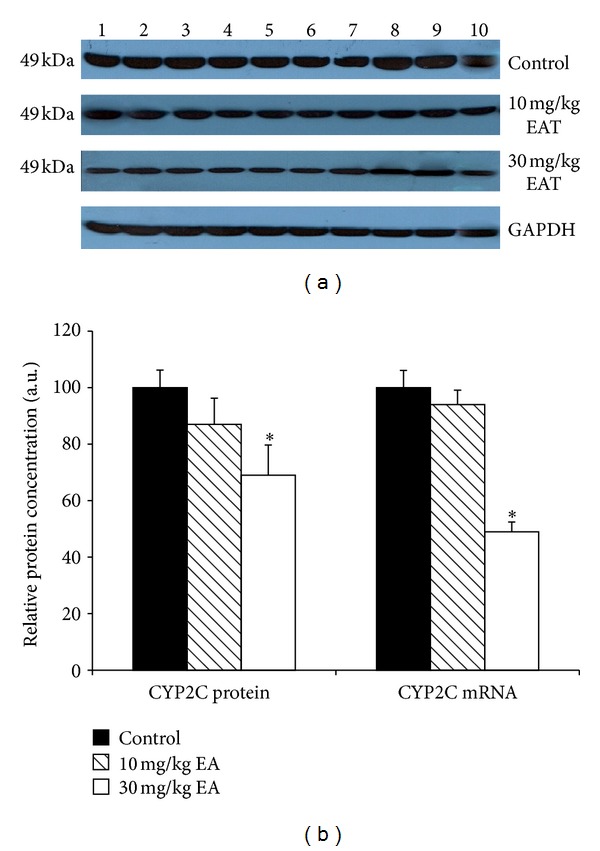
Effect of EA on the expressions of CYP2C protein and mRNA in rat liver microsomes. Rats were treated with EA injection and liver microsomes prepared as described in Section 2. (a) The microsomal proteins were separated by SDS—PAGE, and western blot analysis was performed as described in Section 2. Each lane contained 100 mg microsomal protein. Proteins were detected using chemoluminescent substrate for 3 minutes, and bands were visualized and recorded using a DNR LightBIS Pro Image Analysis System. (b) The expression level of CYP2C mRNAs in control rats and rats treated with EA. Treatments were carried out as described in Section 2. The bar graph represents the mean intensity of the bands obtained from western blot and/or qRT-PCR results. Results are presented as the mean from three independent experiments and expressed as relative mean ± standard deviation. *P < 0.05, compared with the control group.

**Figure 4:**
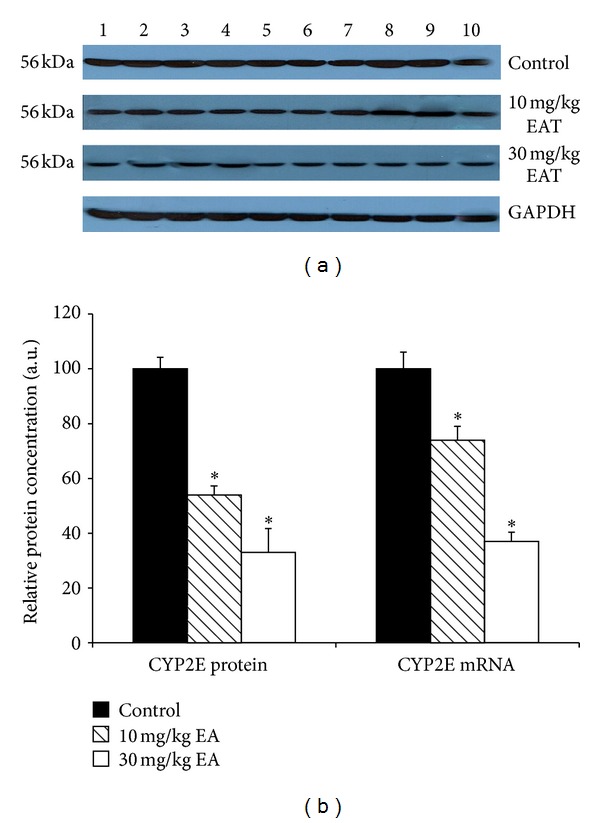
Effect of EA on the expressions of CYP2E protein and mRNA in rat liver microsomes. Rats were treated with EA injection and liver microsomes prepared as described in Section 2. (a) The microsomal proteins were separated by SDS—PAGE, and western blot analysis was performed as described in Section 2. Each lane contained 100 mg microsomal protein. Proteins were detected using chemoluminescent substrate for 3 minutes, and bands were visualized and recorded using a DNR LightBIS Pro Image Analysis System. (b) The expression level of CYP2E mRNAs in control rats and rats treated with EA. Treatments were carried out as described in Section 2. The bar graph represents the mean intensity of the bands obtained from western blot and/or qRT-PCR results. Results are presented as the mean from three independent experiments and expressed as relative mean ± standard deviation. *P < 0.05, compared with the control group.

**Figure 5:**
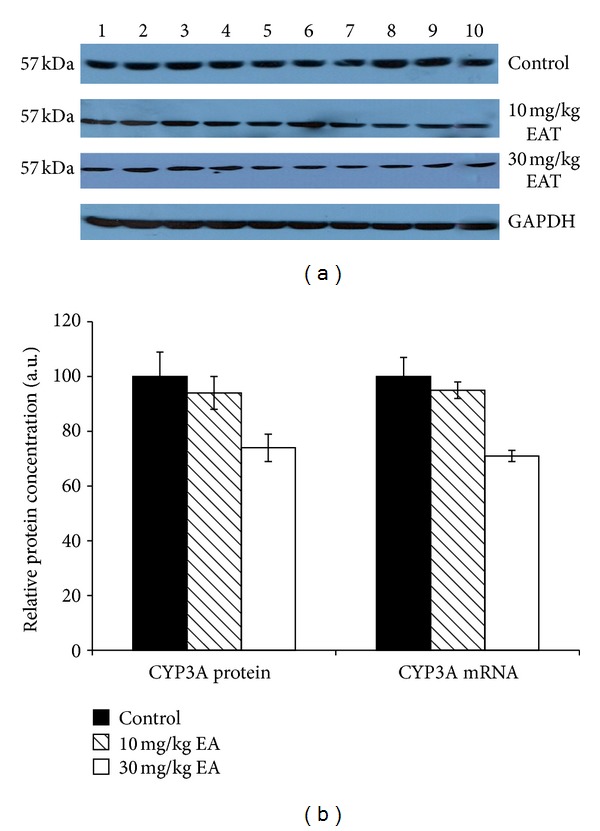
Effect of EA on the expressions of CYP3A protein and mRNA in rat liver microsomes. Rats were treated with EA injection and liver microsomes prepared as described in Section 2. (a) The microsomal proteins were separated by SDS—PAGE, and western blot analysis was performed as described in Section 2. Each lane contained 100 mg microsomal protein. Proteins were detected using chemoluminescent substrate for 3 minutes, and bands were visualized and recorded using a DNR LightBIS Pro Image Analysis System. (b) The expression level of CYP3A mRNAs in control rats and rats treated with EA. Treatments were carried out as described in Section 2. The bar graph represents the mean intensity of the bands obtained from western blot and/or qRT-PCR results. Results are presented as the mean from three independent experiments and expressed as relative mean ± standard deviation.

**Figure 6:**
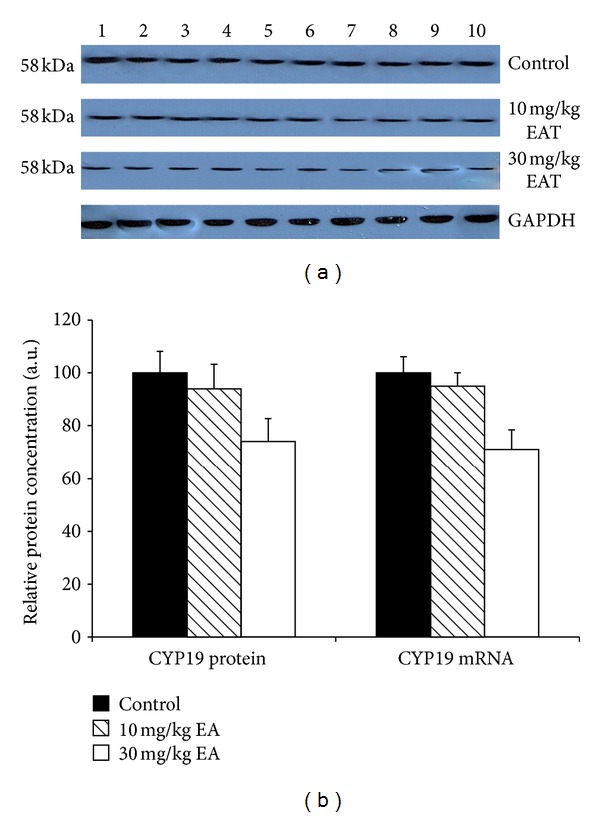
The expression levels of CYP19 protein and mRNA in control rats and rats treated with EA. Treatments were carried out as described in Section 2. Lanes 1–10, rat liver microsome sample. Wells have equal amount of protein. (a) Representative immunoblot analysis of liver microsomal CYP19 proteins in sample and experimental groups, using rabbit anti-rat CYP19 IgG for 1 h at room temperature. Proteins were detected using chemoluminescent substrate for 3 minutes, and bands were visualized and recorded using a DNR LightBIS Pro Image Analysis System. (b) Comparison of CYP19 protein and mRNA levels among experimental groups. The bar graphs represent the mean intensity of the bands obtained from western blot and/or qRT-PCR results. Results are presented as the mean from three independent experiments and expressed as relative mean ± standard deviation.

The activation of catalytic activities was generally consistent with the protein levels of related CYP isoforms in rat liver microsomes that were prepared from control and EA-treated rats (Figures 1–6). The densitometric scanning of western blot results showed that hepatic CYP1A and CYP3A were not significantly changed in the T10 or T30 treated rats relative to the control animals (Figures 1 and 5).

The densitometric scanning of western blot results showed that hepatic CYP2B protein level was decreased 51% and 38% as a result of two different doses of EA treatments. Moreover, T10 and T30 treatments caused a 53% and 32% reduction of the CYP2E protein level (Figures 2 and 4). Although CYP2C and CYP19 protein levels were reduced with T30 treatment, it was not at a significant level (Figures 3 and 6).

The effect of EA on the mRNA levels of CYP isozymes was also determined throughout in this study. The relative CYP1A and CYP3A mRNA levels were not changed significantly in the EA-treated rats as compared to the control animals (Figures 1 and 5). CYP2E level was decreased significantly 79% and 31% in T10 and T30 treated rats, respectively (Figure 4). In addition, CYP2B level was reduced 76% and 39% as a result of two different doses of EA-treated rats relative to the controls (Figure 2). CYP2C level was decreased 49% significantly in EA-treated rats at T30, respectively (Figure 3). Similar to the changes at protein level, CYP19 mRNA was decreased but not significantly (Figure 6). 

## 4. Discussion

Alterations in the cellular metabolism of xenobiotics are the most important mechanisms that play a vital role during chemical-induced carcinogenicity. Therefore, the use of dietary antioxidants is an important preventive method to minimize the pathological and toxic effects associated with xenobiotics [8, 10, 14]. In this context, this study is carried out to examine the dose-dependent effects of EA on specific P450 forms and also on selected phase II and antioxidant enzymes in rat liver.

Plasma AST and ALT, alone or in combination, are primarily recommended for the assessment of hepatocellular injury in rodents and nonrodents in nonclinical studies. They are sensitive markers for drug-induced liver damage, and the elevated activities of these marker enzymes in plasma are indicative of cellular leakage and loss of the functional integrity of cell membranes in the liver [40]. Treatment with EA at different doses (T10 and T30) caused no changes in the activities of these marker enzymes in plasma when compared with the control group. Therefore, EA, at these doses, can be used in preventive or complementary medicine with safety precautions and within the scope of the methods employed in public health.

Among all cytochrome P450 isoforms, CYP1A holds priority due to its role in the metabolism of carcinogens, mutagens, and environmental pollutants. This CYP is the primary P450 involved in the conversion of outstanding carcinogens to the electrophilic metabolites and is known to be induced by its substrates such as benzopyrene. In this study, CYP1A1/CYP1A2-associated enzyme activities, EROD, MROD, and C3ND were all decreased as a result of T30 treatment, consistent with the previous findings of [41]. Although EA treatment resulted in a significant reduction in CYP1A1/CYP1A2-associated enzyme activities, hepatic CYP1A1 protein and mRNA levels were found not to be changed with EA treatments. EA inhibited CYP1A enzymes directly, without an alteration of its gene and protein expression, and the *in vivo* effect depends to a great degree on the concentration of this compound in rat liver. Hence, the results have demonstrated that EA is able to abrogate chemical carcinogenicity most likely by offsetting CYP1A activity and possibly by the scavenging of the electrophilic metabolite.

CYP2E1 is important in the field of toxicology and carcinogenesis, and it also has a role in drug metabolism [42]. It also plays a vital role in the generation of oxidative stress during alcohol-induced toxicity [43]. Intake of the EA both at doses, T10 and T30, for 14 consecutive days decreased A4H and NDMA activities in the liver. In addition, densitometric analysis of western blots showed that the hepatic CYP2E1 protein level was decreased significantly in the EA-treated rats relative to the control animals. Similarly, [44] showed that EA has an inhibitory effect on NDMA. Consistent with protein levels, administration of the EA had also decreased the CYP2E1 mRNA level. Since the regulation of CYP2E1 expression is complex, involving transcriptional, posttranscriptional, and posttranslational events with polymorphism playing a role [45], the observed mRNA decrease resulting from EA treatment could be either transcriptional or posttranscriptional, which remains to be elucidated. Hence, no matter what the mechanism is, EA administration might decrease the carcinogenesis by inhibiting CYP2E1 and reduce reactive oxygen species by affecting the metabolic pathways of alcohol.

Cytochrome 2B, 2C, and 3A enzymes participate in a wide array of metabolism of drugs. Treatment with the EA caused a significant decrease in CYP2B-associated EmND, BPND, BROD, and PROD activities as well as CYP2B protein and mRNA. Moreover, CYP2C-associated APND activity was decreased significantly with T30 dose of EA. Also, CYP2C protein and mRNA level were decreased with the same dose. Similarly, EA-treatment caused inhibitory effect in CYP3A-associated ERND activity in rat liver microsomes. As previously shown with CYP3A or CYP2C activities [46, 47], EA, which is an antioxidant found in large quantities in pomegranate juice, displayed a probably nonmechanism-based inhibitory effect for CYP3A and CYP2C activities in rat liver microsomes. Thus, drug interactions are fairly likely to occur if EA supplements are taken simultaneously with drugs.

The aromatase enzyme, which converts androgen to estrogen, plays a key role particularly in breast carcinogenesis. CYP19-associated enzyme activities, DBFOD, were decreased as a result of T30 treatment. In addition, CYP19 protein and mRNA levels were reduced with T30 to a lesser extent than the activities. Therefore, the observed inhibition resulting from EA treatment could be neither transcriptional nor translational and remains to be elucidated. Taken together with the results of previous reports [20] and the results of the current study, it may be suggested that EA intake may be a viable strategy for the chemoprevention of breast cancer.

Some of the anticancer effects of dietary polyphenols are related, at least partly, to their indirect antioxidant activities [48–51]. For example, the enhancement of GPX, catalase, NQO1, GSTs, and/or phase II enzyme activities by polyphenols could help the detoxification of carcinogenic agents. Hence, the induction of these enzyme activities by flavonoids is possibly associated with cancer chemopreventive effects. In this study, EA treatment caused an increase in GSTs, NQO1, GPX, and CAT activities in rat liver microsomes. Similarly, the induction of liver GSTs, NQO1, and CAT enzyme activities by flavonoids such as genistein, daidzein, flavone, rutin, quercitrin, myricetin, and kaempferol was reported in recent studies [52–56]. These observations may be of importance in view of the potential use of EA both as potent anticancer agents as well as chemopreventive agents.

## 5. Conclusion

In this study, the different doses of EA did not produce the same effects. While the lower dose of EA (10 mg/kg/day) was not effective because its concentration might not have been enough to reach to the site of action and quench all free radicals generated, the higher dose of EA (30 mg/kg/day) was good enough to be effective. In conclusion, the dose-dependent suppression of CYP1A, CYP2E, and CYP19 and the induction of GSTs, NQO1, GPX, and CAT enzymes suggest anticancer as well as chemopreventive roles for EA while reductions in CYP2B, 2C, and 3A explain potential drug interactions.

## Figures and Tables

**Table 1:** Blood serum AST and ALT enzyme activities in control and EA-treated rats.

	AST	Change fold	AST	Change fold
	Unit/min/mg protein		Unit/min/mg protein	
Control	1.53 ± 0.12	—	1.33 ± 0.19	—
10 mg/kg EA	1.55 ± 0.16	—	1.38 ± 0.21	—
30 mg/kg EA	1.54 ± 0.21	—	1.34 ± 0.12	—

**Table 2:** Changes of activities in liver of EA-treated rats.

	Control	10 mg/kg EA	Change (%)	30 mg/kg EA	Change (%)
EROD (pmol resorufin/min/mg prot.)	6.24 ± 2.23	6.53 ± 1.52	—	5.58 ± 3.97	11 ↓
MROD (pmol resorufin/min/mg prot.)	8.39 ± 2.49	8.31 ± 1.07	—	7.33 ± 3.40	13 ↓
C3ND (nmol HCHO/min/mg prot.)	0.40 ± 0.14	0.39 ± 0.02	—	0.24 ± 1.27*	40 ↓
EmND (nmol HCHO/min/mg prot.)	1.96 ± 0.35	1.78 ± 0.85	10 ↓	1.22 ± 0.09*	38 ↓
BPND (nmol HCHO/min/mg prot.)	1.47 ± 0.40	1.29 ± 0.61	13 ↓	0.662 ± 0.308*	55 ↓
BROD (pmol resorufin/min/mg prot.)	2.61 ± 0.86	2.23 ± 1.20	15 ↓	2.03 ± 1.39*	23 ↓
PROD (pmol resorufin/min/mg prot.)	6.51 ± 1.99	5.89 ± 0.907	10 ↓	4.60 ± 2.71*	30 ↓
APND (nmol HCHO/min/mg prot.)	0.339 ± 0.037	0.331 ± 0.031	—	0.154 ± 0.013*	55 ↓
A4H (nmol p-aminophenol/min/mg prot.)	0.344 ± 0.014	0.245 ± 0.08	28 ↓	0.145 ± 0.08*	58 ↓
NDMA (nmol HCHO/min/mg prot.)	0.405 ± 0.01	0.298 ± 0.11	26 ↓	0.114 ± 0.17*	56 ↓
ERND (nmol HCHO/min/mg prot.)	0.117 ± 0.027	0.124 ± 0.021	—	0.085 ± 0.045*	28 ↓
DBFOD (nmol fluorescein/min/mg prot.)	6.66 ± 0.138	6.15 ± 0.427	—	5.12 ± 1.84*	24 ↓
NQO1 (nmol/min/mg prot.)	184 ± 10.25	183 ± 9.49	—	541 ± 23.18*	194 ↑
GST-CDNB (nmol/min/mg prot.)	559 ± 56.71	561 ± 87.11	—	835 ± 13.08*	49 ↑
GST-DCNB (nmol/min/mg prot.)	12.99 ± 4.29	12.90 ± 8.15	—	18.45 ± 3.93*	42 ↑
GST-EA (nmol/min/mg prot.)	10.35 ± 1.61	10.32 ± 1.11	—	13.79 ± 3.22*	33 ↑
CAT (nmol/min/mg prot.)	9.33 ± 0.29	9.25 ± 0.33	—	15.37 ± 0.33*	64 ↑
GPX (nmol/min/mg prot.)	0.048 ± 0.004	0.05 ± 0.007	—	0.103 ± 0.003*	114 ↑

*Significantly different from the respective control value, *P* < 0.05.
